# A Review of Long COVID With a Special Focus on Its Cardiovascular Manifestations

**DOI:** 10.7759/cureus.31933

**Published:** 2022-11-27

**Authors:** Elamein Yousif, Sarah Premraj

**Affiliations:** 1 Internal Medicine, Al Ahli Hospital, Doha, QAT

**Keywords:** post-acute sequelae of covid-19, long term sequelae, covid-19, cardio vascular disease, long-covid

## Abstract

The novel severe acute respiratory syndrome coronavirus 2 (SARS-CoV-2) virus has been the cause of the century’s worst pandemic so far: coronavirus disease 2019 (COVID-19). It has led to unprecedented mortality and morbidity, resulting in devastating consequences worldwide. The acute manifestations of COVID-19 including respiratory as well as multisystem involvement have been causes of great concern among physicians. However, the long-term effects of the coronavirus have left many patients battling with chronic symptoms, ranging from extreme fatigue to cardiomyopathy. In this article, we review the chronic manifestations of COVID-19 with a focus on cardiovascular manifestations. We discuss the pathophysiology, post-acute sequelae, clinical manifestations, approach to the laboratory diagnosis of cardiovascular manifestations of long COVID, and a proposed multidisciplinary treatment method. We also explore the relationship between vaccination and the long COVID syndrome.

## Introduction and background

According to the WHO’s weekly report of coronavirus disease 2019 (COVID-19) on October 30, 2022, over 627 million confirmed cases and over 6.5 million deaths have been reported globally [1]. Although the incidence of new cases is on the decline, the long-term sequelae of severe acute respiratory syndrome coronavirus 2 (SARS-CoV-2) infection have sparked a growing interest among clinicians and researchers. This is due to the fact that survivors of COVID-19 have now been reported to have a higher risk of mortality, and health loss due to a wide spectrum of pulmonary and extra-pulmonary manifestations [2]. In this review, we discuss the definition of post-acute sequelae of COVID-19 (PASC), the epidemiology, pathophysiology, clinical characteristics, and proposed management strategies. Our review touches briefly on the wide spectrum of long COVID while paying special attention to its cardiovascular manifestations.

## Review

Definition of long COVID and epidemiology

The term PASC, otherwise known as long COVID, was officially accepted by the National Institutes of Health (NIH), USA in February 2021, and refers to the persistence of symptoms or sequelae beyond three weeks of SARS-CoV-2 infection onset [3]. Due to the wide spectrum of manifestations and overlapping clinical conditions, there have been various proposed definitions of long COVID. In December 2020, the UK NICE (National Institute of Health and Care Excellence) released guidelines that defined post-COVID syndrome as signs and symptoms that continue for more than 12 weeks after a COVID-19 infection, and are not explained by alternative diagnoses. Long COVID includes both ongoing symptoms (4-12 weeks) and post-acute symptoms (more than 12 weeks) [4].

There are wide variations in the prevalence of long COVID as reported from various countries. In the UK, the latest estimate is 3.3% [5], whereas a study from India placed it at 7.37% [6]. In contrast, the prevalence was reported to be 39% in a study from Denmark and the Faroe Islands [7]. In a recent study published in the USA, the prevalence of self-reported symptoms of long COVID was found to be 13.9% [8]. In a recent meta-analysis, Chen et al. reported a global estimated pooled prevalence of 0.43, with Asia showing the highest figures (0.51), followed by Europe (0.44) and the USA (0.31) [9]. The high variations in prevalence may be attributed to the diversity of the study populations and the differences in the definitions of long COVID [10,11]. Also, those affected during the first wave of the pandemic, when access to testing was limited, may remain undiagnosed, and hence the burden of disease is possibly underestimated.

Risk factors associated with long COVID

In a large cohort study involving more than 480,000 adults from the UK, several risk factors for long COVID were identified. These included female sex, ethnic minority, socioeconomic deprivation, smoking, obesity, and various comorbidities. The study also reported that the presence of comorbidities, such as chronic obstructive pulmonary disease (COPD), anxiety, depression, migraine, multiple sclerosis, fibromyalgia, benign prostatic hypertrophy, erectile dysfunction, and celiac dysfunction, were associated with the development of long COVID [12].

Other reported risk factors are poor pre-pandemic mental health, poor general health, and underlying asthma [13]. With respect to cardiovascular symptoms of PASC, patients with underlying cardiovascular disease (coronary artery disease, hypertension, atrial fibrillation), pre-existing diseases (asthma, diabetes, kidney disease, cancer), and prior hospitalization for COVID-19, have been associated with a higher risk [14]. The exact pathophysiology with respect to risk factors is yet to be understood fully and requires further research.

Pathophysiology

The mechanisms of cardiovascular injury in both acute COVID-19 and PASC have been the focus of recent research. Initial studies have proposed that there is a downregulation of the respiratory and myocardial angiotensin-converting enzyme 2 (ACE2) pathways, resulting in lung edema, myocardial inflammation, and acute lung injury. Additionally, it has been suggested that pro-inflammatory cytokines are upregulated, leading to systemic inflammatory response and multi-organ involvement, especially with cardiovascular compromise [15,16].

The pathophysiologic mechanisms of individual symptoms are multifold and could be explained by basic alterations in physiology. For instance, the possible causes of tachycardia in COVID-19 survivors may include anemia, hypoxia, anxiety, persisting fever, lung or cardiac disease, including SA node dysfunction, myocarditis, and heart failure [17,18].

However, the immunological and cellular-level clues of the pathogenesis of PASC are quite complex. A large study by Su et al. found that certain antibodies such as those targeted against type I interferons could be implicated in the pathogenesis of long COVID. Other mechanisms that are postulated include a high viral load of SARS-CoV-2 at initial diagnosis, co-existence or reactivation of Epstein-Barr virus (EBV) during acute infection, and pre-existing diabetes mellitus [19]. A higher degree of viremia could result in a greater inflammatory response, and hence more end-organ damage. Similarly, it could possibly cause alterations in the immune status, leading to persistent and prolonged organ dysfunction [20]. A cohort study of 215 individuals analyzed the immunoglobulin patterns in both acute COVID-19 infection and up to one year post-infection. The authors discovered a unique immunoglobulin signature, which along with other risk factors such as age, asthma, and pre-morbid illnesses, predicted the risk of developing PASC [21]. From another perspective, Pretorius et al. suggested that the persistence of circulating micro clots in the plasma, which are resistant to fibrinolytic therapy, may explain the lingering symptoms of long COVID [22].

Pathology of Cardiac Complications of Long COVID

In patients hospitalized with acute COVID-19, myocardial injury as evidenced by raised troponins is well documented and has been shown to be associated with a poor prognosis [23]. At the other end of the spectrum, young athletes and previously healthy students, after recovery from COVID-19, have been demonstrated to have subtle myocardial injuries, inflammation, and pericarditis [24,25].

A study involving 346 participants, who were followed up for cardiac involvement from four weeks to four months after COVID-19 infection, showed that those with persistent symptoms had evidence of diffuse myocardial edema on cardiac MRI. The authors concluded that ongoing cardiac inflammation may explain the symptoms such as chest pain, palpitations, breathlessness, and dizziness in patients with long COVID [26]. Mandal et al. have reported high rates of persistently elevated biomarkers (d-dimers and C-reactive protein) for up to eight weeks in patients with breathlessness and cough [27].

In light of these factors, continued systemic and myocardial inflammation appears to be the main pathology for cardiac involvement in PASC.

Symptoms

**Figure 1:**
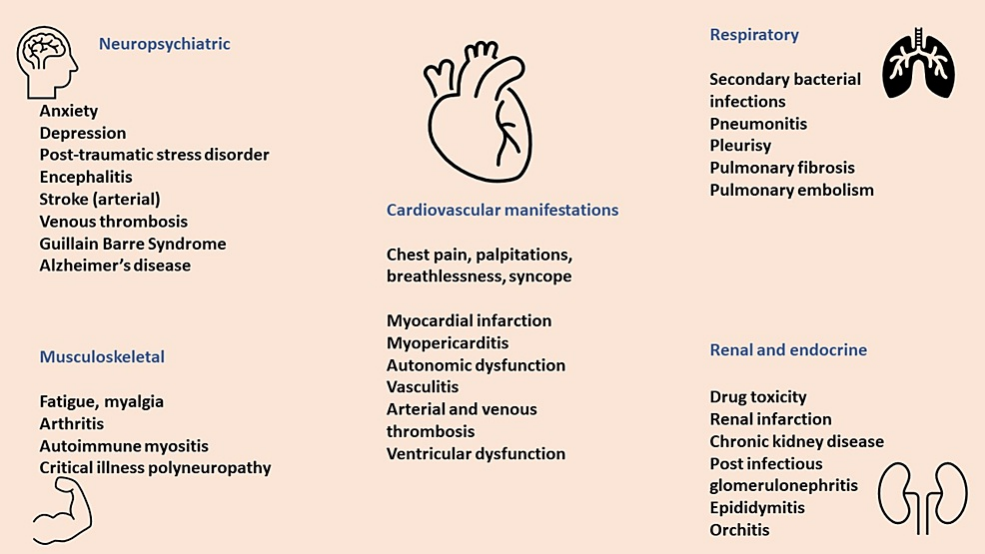
Symptom spectrum of long COVID* *[28]

A constellation of symptoms, spanning various organ systems, is associated with PASC. These are graphically represented in Figure 1. Aside from respiratory system involvement, symptoms have been reported in the cardiovascular, renal, musculoskeletal, gastrointestinal, endocrine, skin, and nervous systems.

The most commonly reported symptoms are fatigue, breathlessness, myalgia, headache, brain fog, loss of memory, and impaired concentration [17]. Virtually every system can be affected by PASC.

Cardiovascular Symptoms

Symptoms specific to the cardiovascular involvement of long COVID include palpitations, chest pain, breathlessness, and postural dizziness with or without syncope. A cross-sectional study, based on patients’ responses to a social media questionnaire in the UK, divided the symptoms into cardiorespiratory and multisystem and found that the former constituted 88.8% of all self-reported symptoms. However, multisystem involvement was found to be associated with more severe disability and functional impairment [29]. Puntmann et al. followed up with patients with mild COVID-19 and assessed them for cardiac symptoms. In their study, 73% of patients reported cardiac symptoms not present before COVID-19. Although the majority had mild or moderate symptoms, 3% of the study group had disabling symptoms. While dyspnea was the most common at 62%, syncope was reported in 3% of patients. They also found that the participants had higher diastolic blood pressure readings as compared to controls [26].

In contrast, a study conducted among medical professionals found that there was no significant difference in cardiac manifestations between the case and control groups at six months, that is, those affected by COVID-19 and healthy individuals had similar clinical features [30].

The difference noted among various studies is probably due to the inhomogeneity in definitions of long COVID. For example, a large systematic review found that studies defined long COVID as ranging from 14 to 110 days post-viral infection, and over 50 different symptoms were identified across 15 studies [31]. Recently, long COVID has been included in the coding of ICD-11 (RA-02). This should result in more uniform reporting in future research.

Diagnosis of long COVID

The diagnosis of long COVID and PASC remains largely clinical. A thorough history taking, physical examination, and review of medical records play a key role in the evaluation of patients reporting lingering symptoms after 12 weeks of infection with SARS-CoV-2. It should be borne in mind that these patients might have already had several visits to doctors across various specialties including general practitioners, internists, pulmonologists, cardiologists, and neurologists, not to mention visits to the emergency department.

There are no specific diagnostic criteria for long COVID. Rather, it is a diagnosis of exclusion, which means that the symptoms cannot be explained by other plausible diagnoses.

With regard to the diagnosis of cardiac symptoms in long COVID, we review each diagnostic modality individually in the following section.

Electrocardiography

The review of electrocardiography (ECG) findings in PASC showed that dynamic changes such as depolarization and repolarization abnormalities were not as frequent in post-COVID patients as in the acute phase [32]. However, sinus arrhythmia and persistent sinus tachycardia have been reported in several studies. Radin et al. have demonstrated both tachycardia and bradycardia, associated with symptoms of dyspnea in wearable device users, up to two to three months following COVID-19 infection [33].

24-Hour Holter Monitoring

Although there is a scarcity of large studies using 24-hour rhythm monitoring in symptomatic post-COVID patients, a few studies in the published literature have shown an increased incidence of arrhythmias including ventricular arrhythmias such as premature ventricular contractions, and non-sustained ventricular tachycardia [34,35].

Echocardiography

Cardiac imaging is a gold standard in the diagnosis of cardiac involvement during acute COVID-19. On follow-up of patients with significant echocardiographic abnormalities such as right ventricular dysfunction, myocarditis, pericarditis, and cardiomyopathy, it was found that most of the pathologies eventually improve. However, ventricular remodeling can occur [36], and a large proportion of patients remain with diastolic dysfunction [37].

Cardiac Biomarkers

Cardiovascular biomarkers (CVB) such as troponin-I, troponin-T, and N-terminal-pro hormone BNP (NT-proBNP) are well known to be elevated in the context of acute COVID-19, indicating acute heart failure, myocarditis, myocardial inflammation, ischemia, and injury. Follow-up of patients with elevated CVB during acute infection showed that a subgroup still had persistently elevated CVB at five months. These were associated with alterations in ventricular function on echocardiography [38].

Cardiac Magnetic Resonance Imaging (CMR)

Several pathological processes such as myocardial edema, hyperemia, necrosis, and fibrosis have been demonstrated in acute COVID-19. CMR plays a central role in the diagnosis of the presence and extent of cardiac involvement. Early studies have demonstrated a high prevalence of cardiovascular injury on CMR, with around 60% of patients showing ongoing myocardial inflammation at 71 days post-recovery from COVID-19 [39]. However, more recent studies have concluded that there is very little evidence for persistent or prolonged inflammation on CMR, even in symptomatic patients with long COVID [40,41]. Baum et al. postulated in their study that a small subgroup of patients with severe functional impairment may be likely to have significant cardiac dysfunction [42].

Other Tests

Certain other tests may also be useful in patients with cardiovascular long COVID, depending on the presenting symptoms and signs.

It is well known that thrombosis is a dreaded complication of acute COVID-19. However, delayed catastrophic pulmonary emboli have also been reported [43]. CT with angiogram has been shown to be useful in detecting delayed arterial thromboemboli as well as microangiopathic diseases [44].

Mancini et al. studied the usefulness of cardiopulmonary exercise testing (CPET) in patients with unexplained dyspnoea post-COVID and showed that it helped to categorize dysfunctional breathing, hypocapnia, and chronic fatigue syndrome with PASC [45]. Autonomic dysfunction is an increasingly recognized manifestation of PASC, presenting as postural dizziness, hypotension, and syncope. Tests such as the tilt table test or 10-minute standing test can prove valuable in the diagnosis of postural orthostatic tachycardia syndrome (POTS) [46].

In summary, a thorough history, clinical examination, and blood test panel including CVBs, ECG, and transthoracic echocardiography at least 8-12 weeks from infection is recommended for those individuals at high risk for cardiac involvement in long COVID. Those with significant abnormalities may be directed for further testing such as CMR, Holter, coronary CT angiogram, or pulmonary angiogram. Referral to specialist clinics (POTS, arrhythmia clinic, psychology support) should be considered where relevant [47].

The management approach for patients with cardiac symptoms of long COVID

Patients with cardiovascular symptoms post-COVID warrant a careful approach, in order to optimize treatment and achieve satisfactory outcomes. The American College of Cardiology Consensus guidelines recommend referral to a specialist cardiologist in the following situations: abnormal cardiac testing results, new or worsening symptoms of underlying cardiac disease, documented cardiac complications during the acute phase of SARS-CoV-2 infection, and persistent or concerning cardiovascular symptoms [47].

There is no proven pharmacological treatment for the cardiovascular symptoms associated with PASC. The management is multidisciplinary and starts with the assessing nurse, who can provide suggestions such as breathing exercises, incentive spirometry, and pulmonary rehabilitation strategies to those with dyspnoea. Other strategies include increasing oral fluids, compression stockings, and behavioral modifications for those suffering from POTS [48].

There is some role for non-steroidal anti-inflammatory agents in the management of pain, in the absence of other contraindications for their use. A graded increase in exercise and return to play is recommended for mild infections. In those with a proven or suspected myocarditis, exercise restriction for three months, followed by gradual resumption is the preferred strategy.

Early referral for mental health and well-being plays a pivotal role in the management of long COVID. Improved access to early care, mental health clinics, rehabilitation, and occupational and social therapy needs to be focused on.

Association between vaccination and long COVID

Vaccination confers proven protection against COVID-19-related complications and mortality. A study from Israel has shown that two doses of vaccination were associated with a lower risk of symptoms of long COVID [49]. Another large study from the UK also found that the post-acute symptoms in infected unvaccinated individuals were more frequent compared to vaccinated ones [50]. Perlis et al., in a study conducted on over 16,000 patients, reported that completion of vaccinations prior to acute infection was associated with a reduced risk for long COVID [8]. It has been proposed that an accelerated viral clearance and an attenuated inflammatory response may, in part, explain the paucity of symptoms following vaccination. Although there have been reports of vaccine-induced myocarditis [51], overall, the risk-benefit ratio seems to be undeniably in favor of vaccination.

## Conclusions

The term Long COVID (or post-COVID) describes a condition characterized by the persistence of symptoms for at least 12 weeks after the onset of COVID-19. The symptoms and signs are clinically varied and involve multiple systems, including the cardiovascular system. The general recurrent symptoms include fatigue, breathlessness, myalgia, headache, loss of memory, and impaired concentration, with several patients reporting a decline in their previous psychophysical performance. Cardiovascular involvement manifests with common symptoms such as palpitations and chest pain, and, less commonly, with events such as late arterial and venous thromboembolism, heart failure episodes, strokes or transient ischaemic attack, and myopericarditis. The diagnostic criteria are mainly based on patient history, and no specific measurable biomarkers or imaging findings have been found so far. Further research is needed to devise a diagnostic tool that can use a combination of clinical symptoms, biomarkers, ECG, and imaging to define cardiac involvement in long COVID.
